# Physical processes controlling the diurnal cycle of convective storms in the Western Ghats

**DOI:** 10.1038/s41598-021-93173-0

**Published:** 2021-07-08

**Authors:** U. V. Murali Krishna, Subrata Kumar Das, Sachin M. Deshpande, G. Pandithurai

**Affiliations:** Indian Institute of Tropical Meteorology, Ministry of Earth Sciences, Pune, 411008 India

**Keywords:** Atmospheric science, Atmospheric dynamics

## Abstract

Diurnal variation of convective storms (CSs) during monsoon season and associated physical mechanisms are significantly important for accurate forecast of short-time and extreme precipitation. The diurnal cycle of CSs is investigated using ground-based X-band radar, Tropical Rainfall Measuring Mission Precipitation Radar, and reanalysis data during the summer monsoon (June–September of 2014) over complex mountain terrain of Western Ghats, India. Diurnally, CSs show a bimodal distribution in the coastal areas, but this bimodality became weak along the upslope regions and on the mountain top. The first occurrence mode of CSs is in the afternoon–evening hours, while the second peak is in the early-morning hours. The diurnal cycle’s intensity varies with location, such that it reaches maximum in the afternoon–evening hours and early morning on the mountain top and coastal areas, respectively. Two possible mechanisms are proposed for the observed diurnal variation in CSs (a) the radiative cooling effect and (b) the surface wind convergence induced by the interaction between land-sea breeze, local topography and large-scale monsoon winds. It is also observed that the CSs developed on the mountain top during afternoon–evening hours are deeper than those along the coast. The higher moisture in the lower- and mid-troposphere, higher instability and strong upward motion facilitate deeper CSs during afternoon–evening hours.

## Introduction

The diurnal cycle of rainfall is one of the most fundamental modes of atmospheric variability^1^. However, it is not completely understood what drives the diurnal cycle of rainfall or how the large-scale atmospheric variability affects the diurnal changes in rainfall, especially over the Indian sub-continent^2^. The mesoscale phenomenon like land-sea breeze, katabatic winds, and orography can modulate the diurnal cycle over different regions^3^. Characterizing the diurnal cycle of CSs will help to understand not only the initiation and evolution process but also the mechanism that drives the local weather and climate. The diurnal cycle of CSs and associated physical driving mechanisms is vital for quantitative precipitation forecast in numerical weather prediction models^4,5^. Analysis of the diurnal cycle in CS frequency and intensity can advance our understanding of different processes related to precipitation variability, boundary layer dynamics, and surface hydrology^6^. The rainfall diurnal cycle is also important to understand the surface energetics and radiation budget^7^. It also plays a vital role in the simulation of non-linear feedback processes between convection and radiation in the global climate models. For instance, Schlemmer and Hohenegger^8^ suggested that the accurate characterization of the cold pool is essential to represent more accurately the diurnal cycle of precipitation in global models. Therefore, the diurnal variation in CSs over different geographical regions gained much attention during recent past^9–16^. Although the significance of the diurnal cycle of CSs has been widely recognized, understanding the diurnal cycle of CSs and associated physical mechanisms is still challenging, especially over mountainous region like Western Ghats (WG).

The prediction of amplitude and phase of the rainfall diurnal cycle is very challenging^17,18^. The diurnal cycle of convection is poorly represented by cumulus parameterization schemes in weather and climate models^19,20^. The numerical models produce the rainfall peak near local solar noon, which is several hours earlier than observations^21^. The misrepresentation of convective rainfall’s diurnal timing is the major source of uncertainty in the rainfall forecast over the tropical regions^22^. This model bias can also generate errors in radiation balance at the surface. The proper representation of the interaction between various elements of atmosphere-land-ocean system in numerical weather and climate models can reproduce the same phase and amplitude of the observed diurnal cycle of clouds and convection^23,24^. In addition, orography can affect the propagation of convective systems^25^, which can further delay the onset of convection over a particular region. Although several mechanisms could lead to pre-onset of convection in the numerical models, an improved understanding of the physical processes that control the diurnal cycle of CSs is important for advancing weather and climate models to minimize model biases in forecasting the convective and extreme rainfall.

WG’s rainfall is generally affected by underlying complex topography, monsoon flow, and land-sea contrast^4^. The topography of WG plays an important role in the spatial distribution of rainfall. For instance, the rainfall increases along the windward side and decreases at the WG’s leeward side^26,27^. Using satellite and reanalysis data over the Asian monsoon region, Hoyos and Webster^28^ showed that the rainfall peak occurs over the oceans (offshore regions) due to the mountain’s upstream effects but not over the land regions. Nesbitt and Anders^29^ found a steep west to east gradient in the WG’s mesoscale precipitation patterns. Shige et al.^12^ observed that the rainfall maximum is concentrated along the WG upslope regions compared to the offshore locations, as observed in previous studies. Further, deep moist convection is related to the underlying topography^30^. In general, the rainfall intensity is higher along the windward slopes of the mountains^31^. Medina et al.^32^ showed that the upslope flow at the mountain could develop deep convection with higher rainfall intensities. Zhang and Smith^33^ studied the physical processes that control the monsoon precipitation over WG using the model simulations. They showed that the convective rainfall along the coastal regions is forced by the topography and diurnal heating in the WG. These studies highlighted the importance of WG topography in the spatial distribution of rainfall.

The availability of high spatial and temporal resolution measurements, especially from remote sensing platforms, provide an opportunity to study the diurnal variation of CSs worldwide^14,30,34^. Pronounced spatial and temporal inhomogeneity is present in the characteristics of the diurnal cycle. For example, Basu^3^ noticed a different diurnal cycle of precipitation over the land, ocean, and orographic regions using satellite measurements and model forecast data. Raut et al.^35^ studied the diurnal variation in the cumuliform clouds over the Indian subcontinent during the summer monsoon. They found an early-morning maximum in the cloud occurrence over the Arabian Sea. Using Tropical Rainfall Measuring Mission (TRMM) Precipitation Radar (PR) data, Romatschke and Houze^9^ found a double peak structure with afternoon and early-morning maxima in the occurrence of convection over the Indian west coast. They showed that the afternoon maximum is related to convection over land and the morning peak with the convection over the ocean. A weak diurnal cycle of rainfall with a peak in morning hours is observed along India’s west coast^36^. Shige et al.^12^ studied the effect of the diurnal cycle and orography on the intraseasonal oscillations in the summer monsoon rainfall over the WG. They found a continuous rainfall with a slight nocturnal and afternoon–evening maximum over the WG region. Utsav et al.^37^ from radar observations showed that the CSs exhibit a bimodal distribution with afternoon and nocturnal maxima. They also observed an eastward propagation of CSs from coastal regions through windward side to the WG’s leeward sides. The above studies emphasized a significant diurnal cycle in the rainfall over WG and surrounding oceanic areas. However, the mechanism/factors controlling the observed diurnal cycle of rainfall is still lacking, which need greater attention in context of orography. This motivated us to investigate the diurnal cycle of CSs and their underlying processes over WG’s complex terrain. This study explores the following scientific objectives by taking X-band radar observations and reanalysis data:

i. How does the diurnal cycle of CSs affected by the underlying topography?
ii. The driving mechanisms (dynamical and thermodynamical processes) that control the observed diurnal cycle of CSs over the complex topography.
iii. The factors controlling the development of shallow CSs along coastal regions, and deeper CSs over the mountain top.

**Figure 1:**
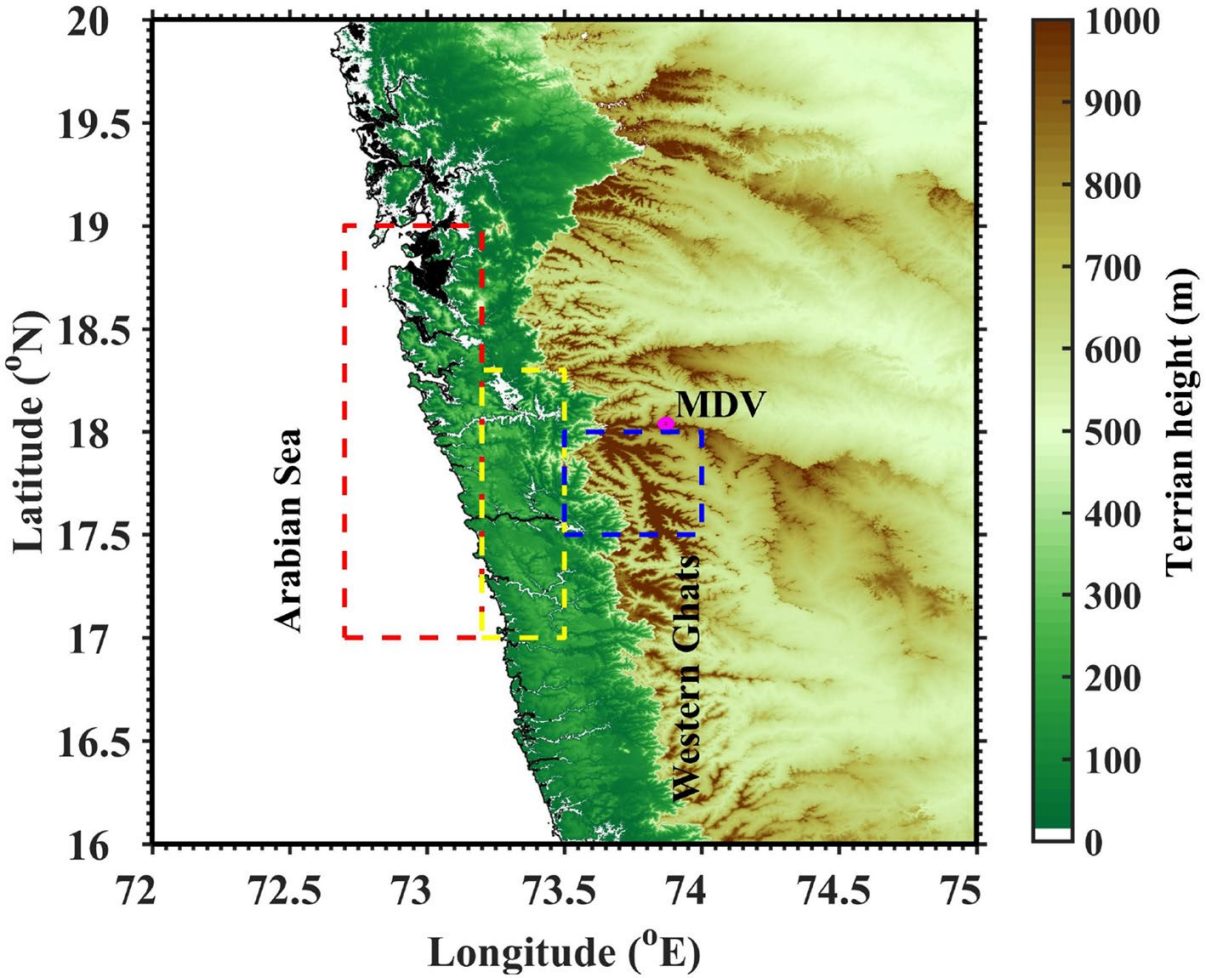
Topographical map of the study domain generated using the Shuttle Radar Topography Mission (SRTM) Version 3.0 Global 1 arc second data^60^ (https://earthdata.nasa.gov/learn/articles/nasa-shuttle-radar-topography-mission-srtm-version-3-0-global-1-arc-second-data-released-over-asia-and-australia). X-band radar location, Mandhardev (MDV), is shown with a solid magenta circle. The coastal, windward and high altitude regions are shown with red, yellow, and blue dashed rectangles, respectively. The topographical map is generated using MATLAB R2019b programming language.

**Figure 2:**
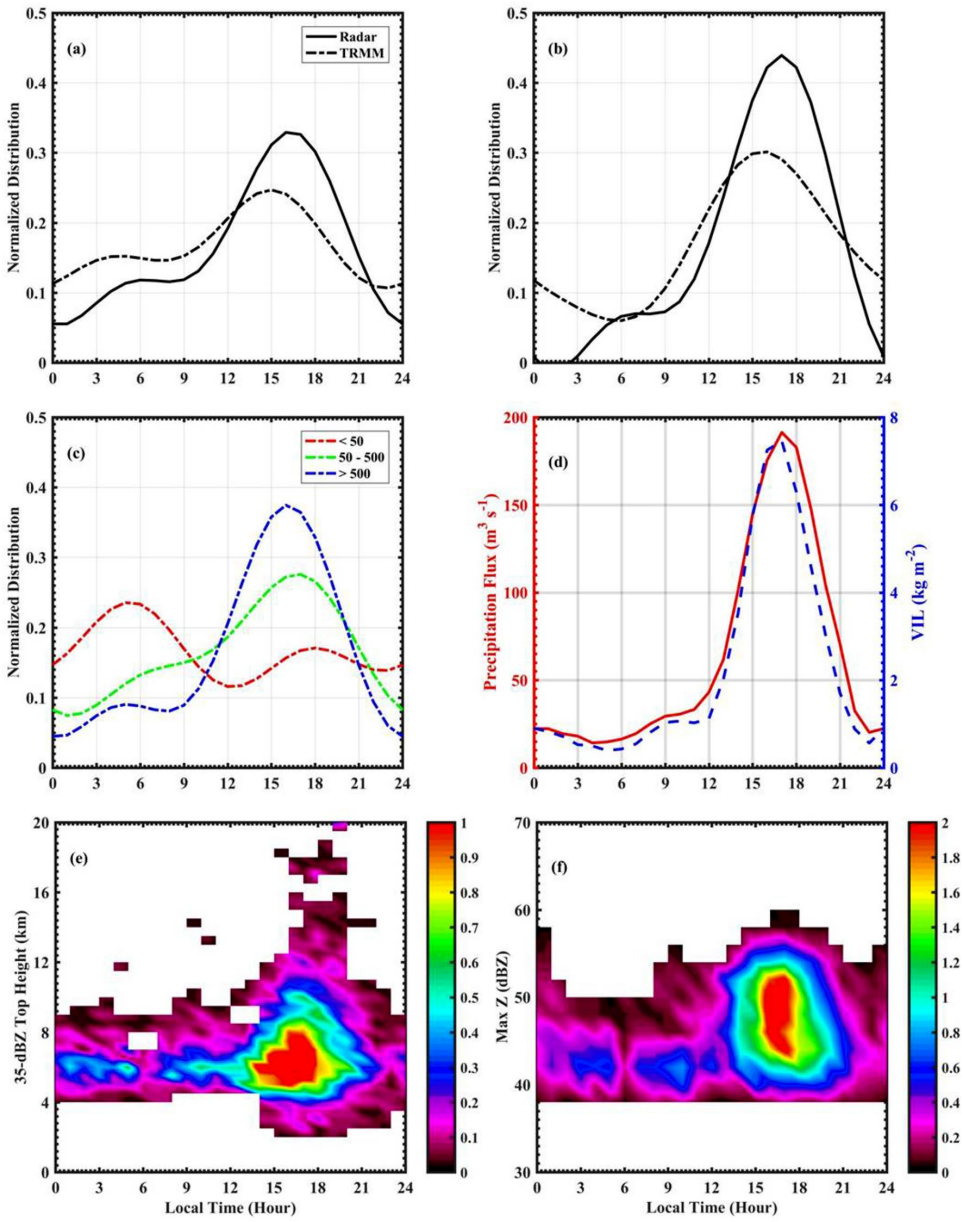
The diurnal cycle of CS occurrence on (**a**) windward and (**b**) leeward sides of radar using X-band radar (solid-line) and TRMM-PR (dashed-line) observations, (**c**) with the topography (height in meters) on the windward side of radar. (**d**) The diurnal cycle of average precipitation flux and VIL over the radar domain. Two-dimensional histograms of (**e**) 35-dBZ echo top height (ETH), (**f**) maximum reflectivity. Here, the colorbar represents normalized occurrence.

**Figure 3:**
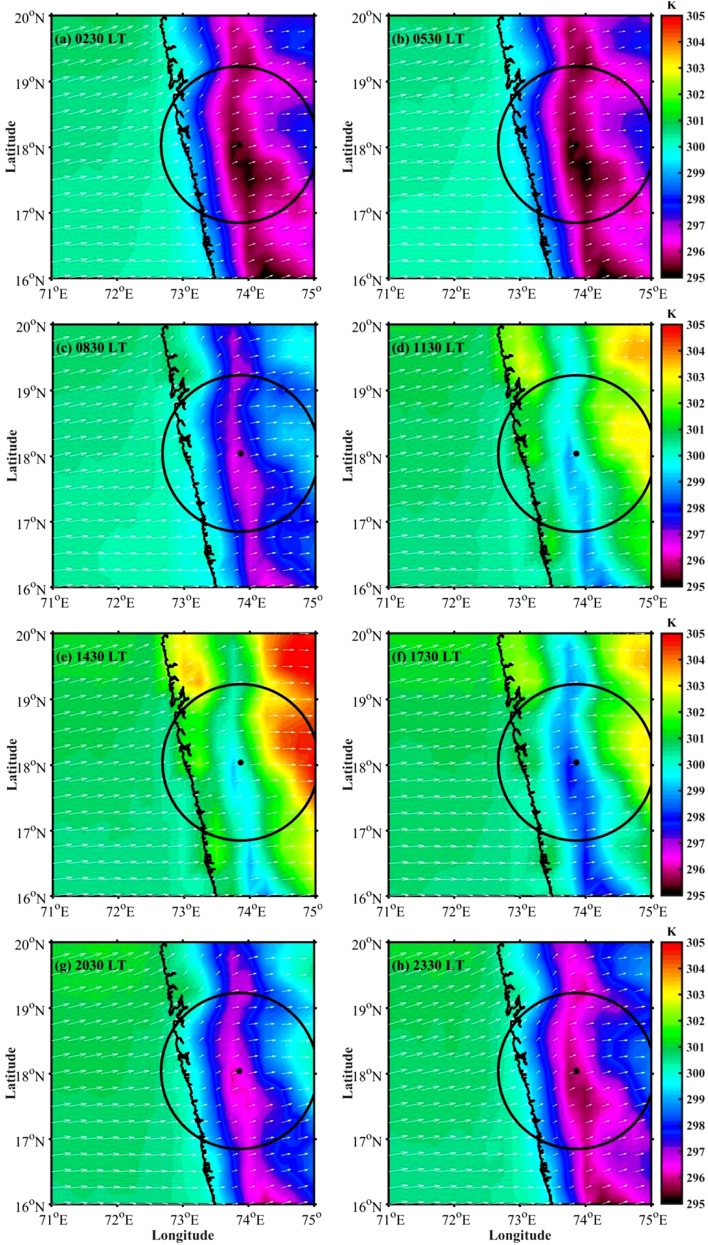
Spatial distribution of mean near-surface temperature (color-filled contours) and wind vector (arrows) during (**a**) 0230 LT, (**b**) 0530 LT, (**c**) 0830 LT, (**d**) 1130 LT, (**e**) 1430 LT, (**f**) 1730 LT, (**g**) 2030 LT, and (**h**) 2330 LT from the ERA5 reanalysis data during summer monsoon (JJAS). The solid black dot represents radar location, and black circle represent radar surveillance domain.

## Results and discussion

### Diurnal cycle of CSs

This sub-section presents the diurnal cycle of CSs observed using X-band radar measurements over WG during the Indian summer monsoon period (JJAS). The topography map of the WG and radar site is shown in Fig. 1. The possible mechanisms for the observed diurnal cycle are explored using high-resolution hourly data from ERA5.

Figure 2a,b shows the diurnal cycle of CSs frequency on the windward and leeward sides of the radar domain respecitvely from X-band radar and TRMM-PR measurements. TRMM-PR observations for 11-years are considered over the radar domain to calculate the CSs frequency. A bimodal distribution in CS occurrence is observed in the X-band radar measurements on the mountain’s windward side (Fig. 2a). There is a primary peak in CS occurrence in the afternoon hours and a secondary maximum in the early-morning hours. Our finding supports the results of Shige et al.^12^, where they observed a bimodal distribution in the rainfall pattern over WG. The CS occurrence starts increasing in the midnight to early-morning hours at about 0100–0600 LT (LT = UTC + 0530 h). The CS occurrence decreases slightly between 0600 and 0900 LT and then increases rapidly to its maximum in the afternoon–evening at 1600–1800 LT. The TRMM-PR also shows the bimodal distribution with a primary maximum in the afternoon–early evening and a secondary maximum in the early-morning hours. However, the maximum CS frequency is shifted ($\sim$ 1 h) in the TRMM-PR measurements. The peak occurrence shift may be due to different sampling intervals ($\sim$ 12 min volume scan of X-band radar and instantaneous scan of TRMM-PR), different horizontal resolutions, viewing aspects, propagation paths, resolution volume size, and sensitivity between X-band radar and TRMM-PR measurements^38^. Krishna et al.^39^ compared the diurnal cycle in rainfall measured from impact disdrometer and TRMM along WG’s windward side. They also observed a bimodal distribution in the rainfall over WG. Johnson et al.^40^ observed a bimodal distribution of orographic rainfall with maxima during afternoon–evening and near midnight hours in the Northern America. Similarly, from micro rain radar observations, Endries et al.^41^ found two rainfall maximum, one in the afternoon and the other in mid-night over the tropical Andes. On the leeward side, the CS frequency shows bimodal distribution with a strong maximum in the afternoon–evening hours. The TRMM-PR could capture the afternoon–evening maximum, however, the peak occurrence is shifted by $\sim$ 1 h. The intensity of the diurnal cycle is stronger on the mountain leeward side compared to the windward side. This finding is consistent with Shige et al.^12^ that the diurnal cycle of rainfall is weak along the windward side, and the diurnal cycle with higher amplitude is in the leeward sides of WG. Shige et al.^12^ analyzed 16 years of TRMM-PR data to understand the effect of orography on the diurnal cycle of rainfall over WG. They showed that the diurnal cycle of rainfall is a function of ambient upstream wind. Under strong wind regime, the mechanically forced circulation dominates, and hence a continuous rainfall with a small double-peak structure in afternoon–evening and early-morning are observed on the coastal and windward side of WG. Whereas in the leeward side, a rain shadow region, the convection is dominated by the diurnal cycle of surface heating^42,]^^43^. Hence, the CSs diurnal cycle is stronger on the leeward side than on the coastal and windward sides.

The diurnal cycle of CSs with the topography of WG is shown in Fig. 2c. Here, the topography of WG is classified into three regions based on mean sea level (MSL) heights: coastal and oceanic (MSL height < 50 m), upslope (upwind) region (MSL height between 50 and 500 m), and higher altitudes (MSL height > 500 m). The temporal distribution in CSs varies with the topography. In addition, the propagation of CS from coastal and oceanic regions to high altitudes is also evident. A prominent bimodal distribution in the CSs occurrence can be found in the coastal and oceanic regions, however, this bimodal distribution is absent along upslope regions of the WG. The CSs occurrence is highest over coastal and oceanic areas in the early morning hours (0300–0600 LT), whereas it is highest in the afternoon–evening hours (1400–1800 LT) over the mountain top. The CS occurrence is intermediate during afternoon–evening hours along the upslope regions. These results are similar to those observed during the 2004 North American Monsoon Experiment (NAME). Using S-band and C-band radar observations during the NAME, Lang et al.^44^ studied the diurnal cycle of rainfall over different topographic regions. They observed that the rainfall maximum occurred during afternoon–evening time at high altitudes of the Sierra Madre Occidental, and midnight to early-morning hours over coastal plains and the Gulf of California. Further, it can be observed that the time of afternoon–evening maxima is different in different topographic regions of WG. The maximum occurrence in CS can be observed during 1500–1600 LT over higher altitudes, at about 1700 LT over the upslope regions, and at about 1800 LT in the coastal regions. Several studies documented the diurnal cycle of rainfall over WG^9,12,36^, however, the effect of topography on the diurnal cycle is unexplored. The physical processes responsible for the observed diurnal cycle are yet to be documented, especially over complex topography like WG. The afternoon–evening peak can be related to the diurnal change in incoming solar radiation over the high terrain. The physical mechanisms behind the nocturnal maximum in the CSs occurrence are worth investigating. The nighttime peak can be induced by several processes such as convergence between nighttime downslope wind and the prevailing monsoon flow^9^. The possible mechanisms responsible for the afternoon and early morning peak in the CSs occurrence are discussed later.

The diurnal cycle of precipitation flux and vertically integrated liquid water (VIL) over the radar domain is shown in Fig. 2d. The precipitation flux represents the intensity of rainfall and VIL measures the integral of liquid water in a vertical column of the storm. It is observed that the precipitation flux and VIL shows a maximum during afternoon–evening hours, consistent with CS frequency. This indicates that the CSs are more intense during afternoon–evening hours. To understand the diurnal variation in CSs intensity, 35-dBZ echo top height and maximum reflectivity are examined in Fig. 2e,f. The 2D histogram of 35-dBZ echo top height (Fig. 2e) indicates deeper CSs during afternoon to evening hours. It is observed that the occurrence of CSs is in general higher during afternoon to evening time. The histogram of maximum reflectivity (Fig. 2f) indicates that the CSs present during afternoon to evening hours are more intense than other time. Generally, the intense rainfall occurs in the afternoon–early evening hours over the land regions^45^. In the mountainous region of northwestern Mexico, Johnson et al.^40^ showed that the convection is initiated by late-morning hours and developed to deep convection in the afternoon–evening time.

### Plausible mechanism for the observed diurnal cycle of CSs

It is known that the CSs initiate over the ocean in the morning (0830–1130 LT) and propagate eastward to upslope regions to mountain peak (1130–1430 LT), where it reaches the maximum at about 1430–1730 LT, and then move to leeward side^37^. The diurnal variability in CSs may be due to the diurnal cycle of solar radiation and moisture^15,46^, air–sea interactions^47,48^, or large-scale atmospheric conditions^10,14^. To better understand what drives the diurnal cycle in CSs occurrence over the WG, this sub-section presents the diurnal variability of large-scale atmospheric conditions like temperature, moisture, and winds etc. To examine the plausible mechanism for the diurnal cycle of CSs, 3-hourly winds and temperature at 0230, 0530, 0830, 1130, 1430, 1730, 2030, and 2330 LT are analyzed using the ERA5 dataset. Figure 3a–h shows the 3-hourly near-surface winds (10 m) and temperature (2 m) over the radar domain. There is a large diurnal variation in near-surface temperature over land. The near-surface temperatures are relatively lower during nighttime (Fig. 3a,b) and higher at 1430 LT (Fig. 3e) over land. There is no significant diurnal variation in near-surface temperature over the ocean (Fig. 3a–h), which varies between 298 and 304 K. The mean prevailing near-surface winds are south-westerly throughout the day during the monsoon season. The wind speed over the ocean is much higher than the winds over land throughout the day. Over the land regions, the prevailing westerlies are stronger during afternoon hours compared to nighttime.

**Figure 4:**
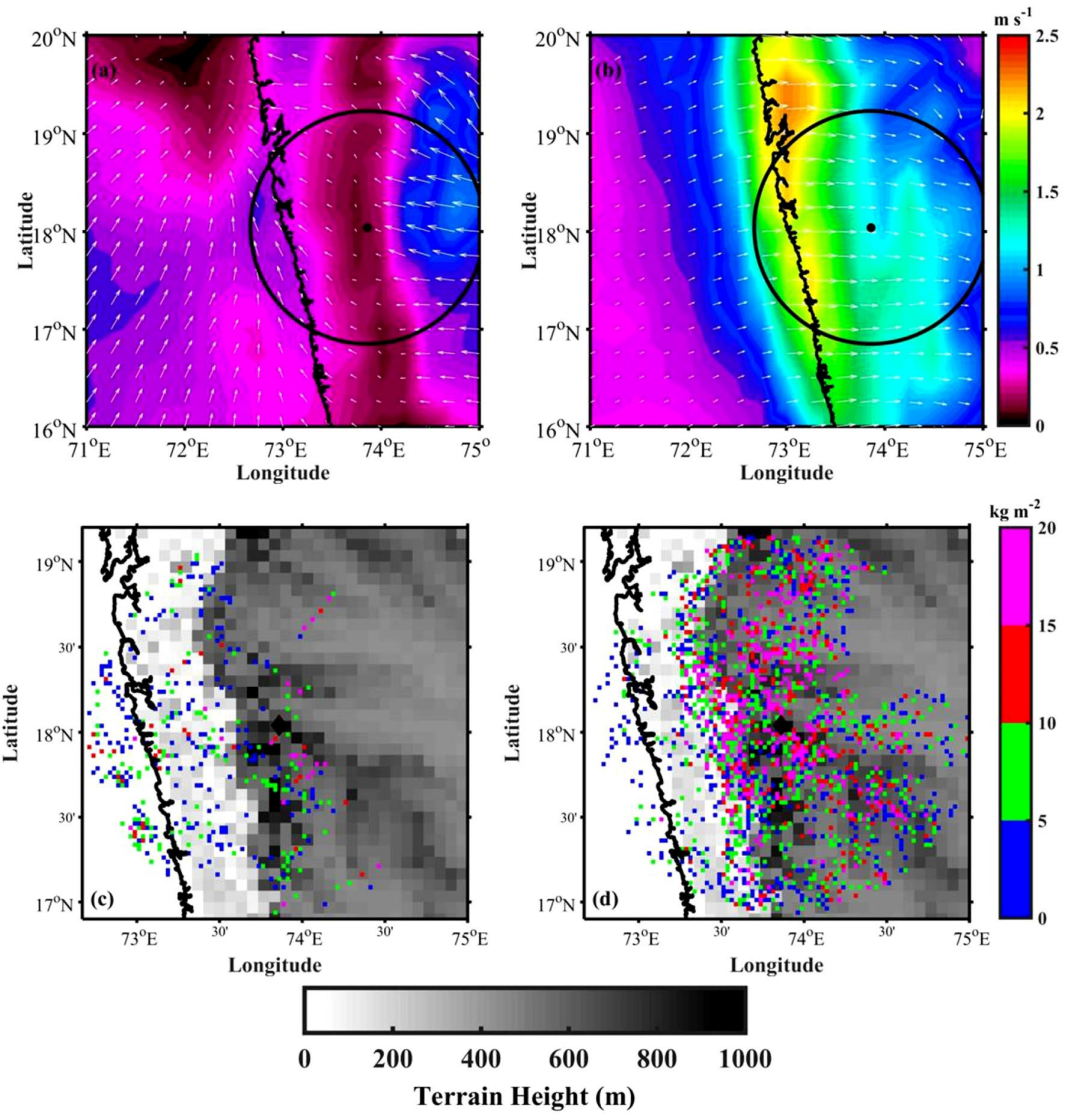
The wind speed (colour-filled contours) and wind vector differences (**a**) between 0230 and 2030 LT, (**b**) between 1430 and 0830 LT. The VIL difference between (**c**) 0000–0600 and 1800–2400 LT, (**d**) 1200–1800 and 0600–1200 LT. The radar domain topography is considered in (**c**,**d**) and shown with graycolor scale.

The diurnal change in the near-surface winds can be better represented by considering the differences between night and day time periods viz. between 0230 and 2030 LT, and between 1430 and 0830 LT. Figure 4a,b shows the wind speed and wind vector differences between 0230 and 2030 LT, and between 1430 and 0830 LT, respectively. Between 0230 and 2030 LT (Fig. 4a), the wind difference is mostly offshore and the temperature over land is significantly lower than ocean (Fig. 3a and Fig. 3g), indicating that the wind flows from mountain to coastal and oceanic regions during nighttime. This indicates the development of land breeze/downslope winds at night due to the radiative cooling. It is observed that the negative vertical velocities (in pressure coordinates) and convergence in the low-levels during 0230 LT over the ocean (Figure not shown), indicating the active convection during nighttime over the oceanic region. So, the development of land breeze/downslope winds in response to the land-sea temperature contrast is responsible for the observed nocturnal maxima over oceanic regions. On the other hand, between 0830 and 1430 LT, westerlies get strengthened as they encounter the WG mountain and produce uplift (Fig. 4b). It is interesting to note that near 180$^\circ$ change in winds at about 12 h apart. This is due to the continental diurnal heating that provides energy to the monsoon circulation^49^. Figure 4c shows the VIL difference between 0000–0600 and 1800–2400 LT over the radar domain (note the difference in the axes limits between Fig. 4a,c). It can be observed that comparable larger data points are present along the coastal regions in the VIL difference. This further indicates the development of CSs over coastal regions in the early-morning hours. This could be due to the convergence of downslope winds from the mountain top with the monsoon flow during nighttime. In addition, the VIL difference is low-to-moderate (<10 kg m^−2^), indicating smaller amount/less intense convection during nighttime. During afternoon hours, the WG mountain serves as an elevated heat source. This can induce the mountain upslope flow, producing maximum occurrence in the CSs. The wind vector difference between 1430 and 0830 LT (Fig. 4b) is strongly onshore, induced by the diurnal temperature contrast between land and ocean. This suggests the development of sea breeze during the afternoon hours. Increased zonal winds along the WG mountain during afternoon hours are evidenced in the Weather Research and Forecasting (WRF) model simulations by Flynn et al.^2^. They relate this finding to the thermal gradient and mixing down of high momentum air in the boundary layer. The VIL difference between 1200–1800 and 0600–1200 LT (Fig. 4d) is high over higher altitudes. The high amount of VIL difference on the mountain top indicates higher/intense convection during afternoon hours. This may be due to the interaction of monsoon flow with the local topography.

**Figure 5:**
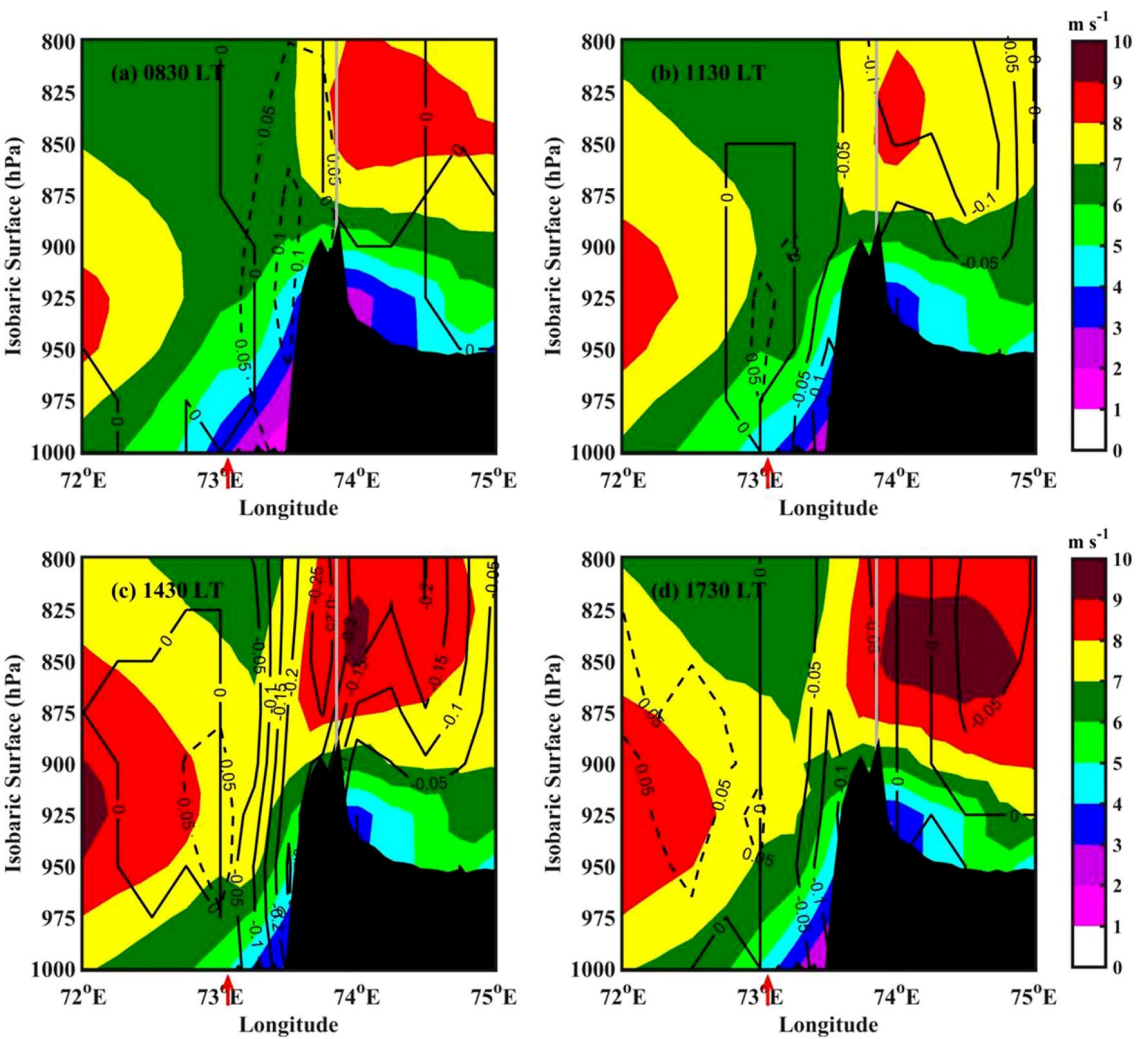
The mean wind speed (colour-filled contours) and vertical velocity (in pressure coordinate) deviation from the mean (black contours) along 18$^\circ$ N during (**a**) 0830 LT, (**b**) 1130 LT, (**c**) 1430 LT, and (**d**) 1730 LT. The solid contours represent negative vertical velocity (upward motion), and dashed contours represent positive vertical velocity (downward motion). The coastline is indicated with a red colour arrow below the X-axis. The topography along 18$^\circ$ N is shown by black shading.

**Figure 6:**
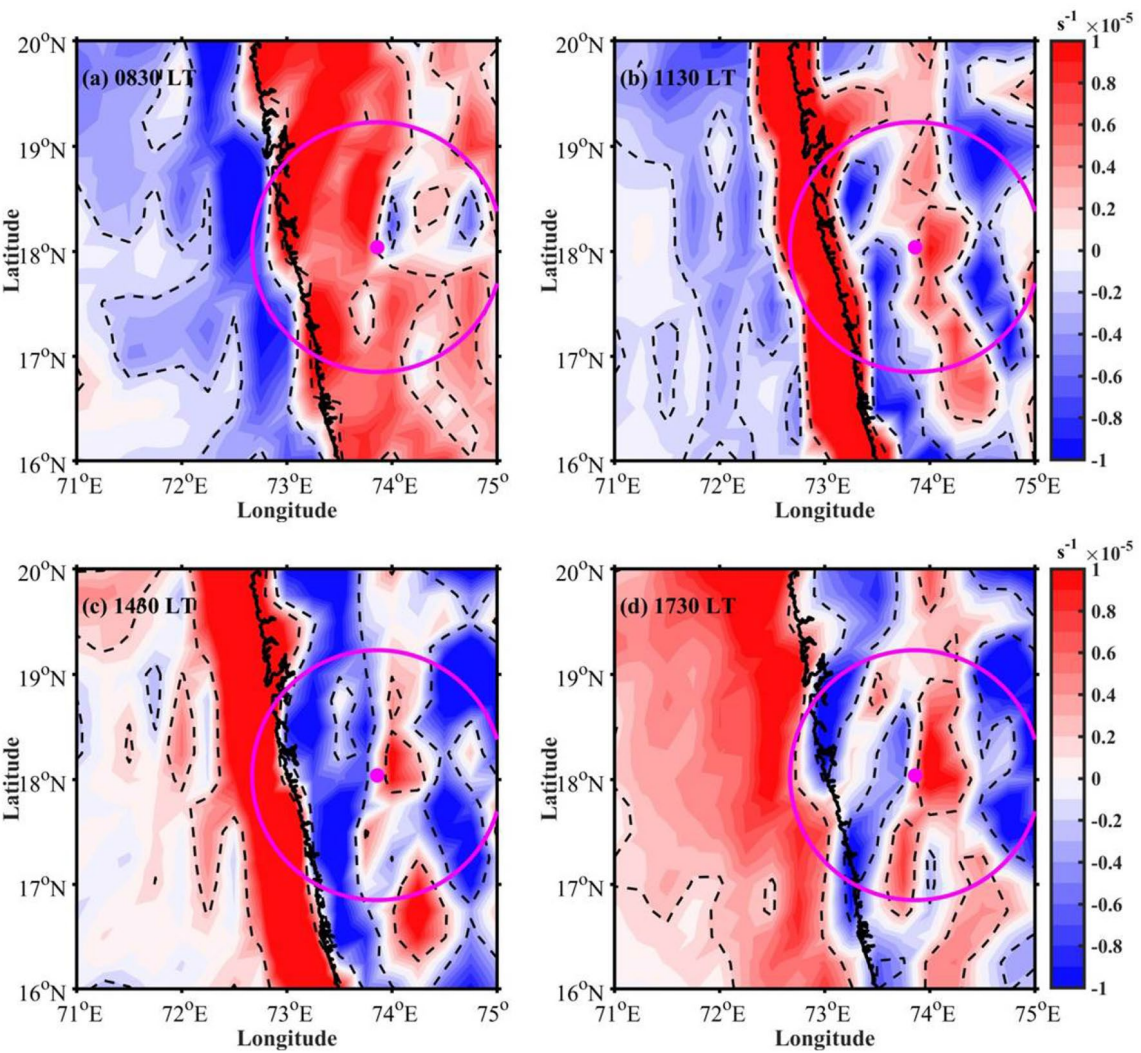
Spatial distribution of mean removed divergence at (**a**) 0830 LT, (**b**) 1130 LT, (**c**) 1430 LT, and (**d**) 1730 LT. The data significant above 90$\%$ confidence level are shown with dashed contours.

The vertical cross-section of deviation in vertical velocity from the mean and perturbation in horizontal winds at 3-h interval along 18$^\circ$ N (which is roughly normal to the coast) is shown in Fig. 5. The location of the coastline is shown with a red colour arrow below the X-axis. The average prevailing winds are south-westerly (onshore) over the radar domain (Figure not shown). At 0830 LT (Fig. 5a), significantly weakened flow (colour-filled contours) can be found from the surface to 950 hPa level beyond 72.5$^\circ$ E longitude. This could be due to the downslope winds (Fig. 4a) coming from the mountains. The vertical motion deviations (contours) are negative over coastal and oceanic regions during 0830 LT, indicating updrafts during this time. The spatial distribution of deviation in surface divergence from the mean (Fig. 6a) is significantly negative over the ocean at 0830 LT, indicating the surface convergence. So, it is evident that the downslope winds converge with the strong ambient wind near the coastal mountain regions and promote updrafts. Thus, the downslope wind plays a vital role in developing CSs in the oceanic region during morning time. Using WRF model simulations, Flynn et al.^2^ studied the diurnal cycle in rainfall over WG during monsoon seasons of 2009–2010. Their study revealed that the land breeze during early-morning hours supports the rainfall maximum along the offshore regions. With the radar observations and reanalysis data, Chen et al.^10^ showed that land breeze is responsible for the nocturnal rainfall maximum in China’s Pearl River Delta region during the Mei-Yu season. More recently, Zhu et al.^15^ noted the multiscale interactions between large-scale circulations and land-sea breeze that plays a vital role in the diurnal cycle of rainfall over the tropical islands.

With increased solar radiation in the morning hours, the horizontal wind speed increases at the coastal and oceanic regions during 1130 LT (Fig. 5b). At this time, negative vertical velocity and convergence (Fig. 6b) can be found over coastal and upslope regions. This shows that the CSs are propagated eastward to coastal mountain regions by 1130 LT. At 1430 LT, the vertical motion deviations are negative over higher altitudes (Fig. 5c). The surface convergence (Fig. 6c) is also negative along the upslope regions and on the mountain peak, indicating further propagation of CSs towards the mountain peak by afternoon. With the maximum solar heating in the afternoon, the rising motion induced by the low-level convergence between the sea breeze front/seasonal winds and the mountain enhances. This indicates that the orographic response to the prevailing monsoon flow from the surrounding oceanic regions superimposed with the land-sea thermal contrast is responsible for the afternoon maximum in the CSs occurrence over high altitude regions in the WG. Romatschke and Houze^9^ observed the afternoon maximum in the rainfall over the Indian west coast. The present analysis supports the mechanism hypothesized by Romatschke and Houze^9^ in explaining the diurnal cycle of rainfall over the WG region. At 1730 LT, the negative vertical motion deviations (Fig. 5d) and convergence (Fig. 6d) can be observed along the coastal and leeside of the mountain, however, the magnitude is small. This may be due to the dissipation of CSs and further propagation to leeside by evening.

**Figure 7:**
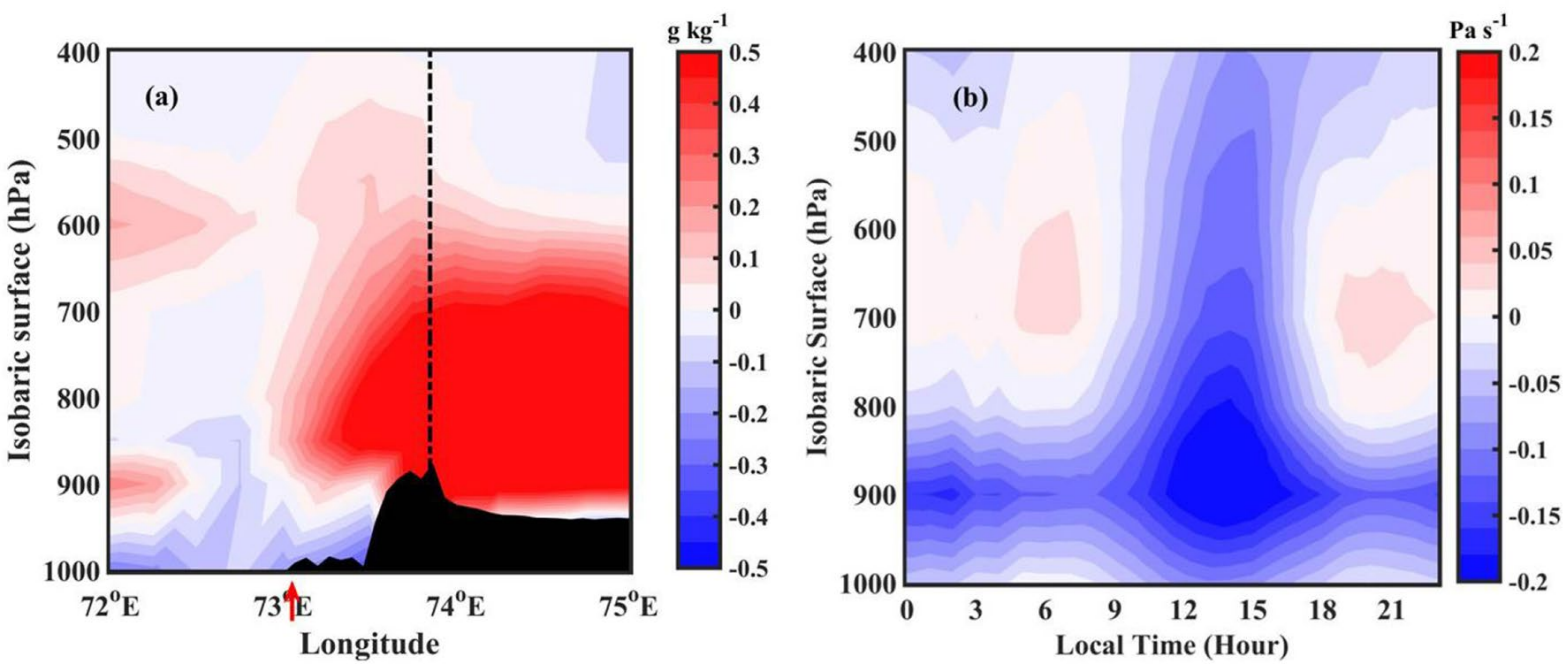
(**a**) Longitude-height (in pressure coordinates) cross-section of specific humidity difference between afternoon (1200–1500 LT), and nighttime (0000–0300 LT) over radar domain. (**b**) Time-height (in pressure coordinates) cross-section of omega (vertical velocity in pressure coordinates; Pa s^−1^).

**Figure 8:**
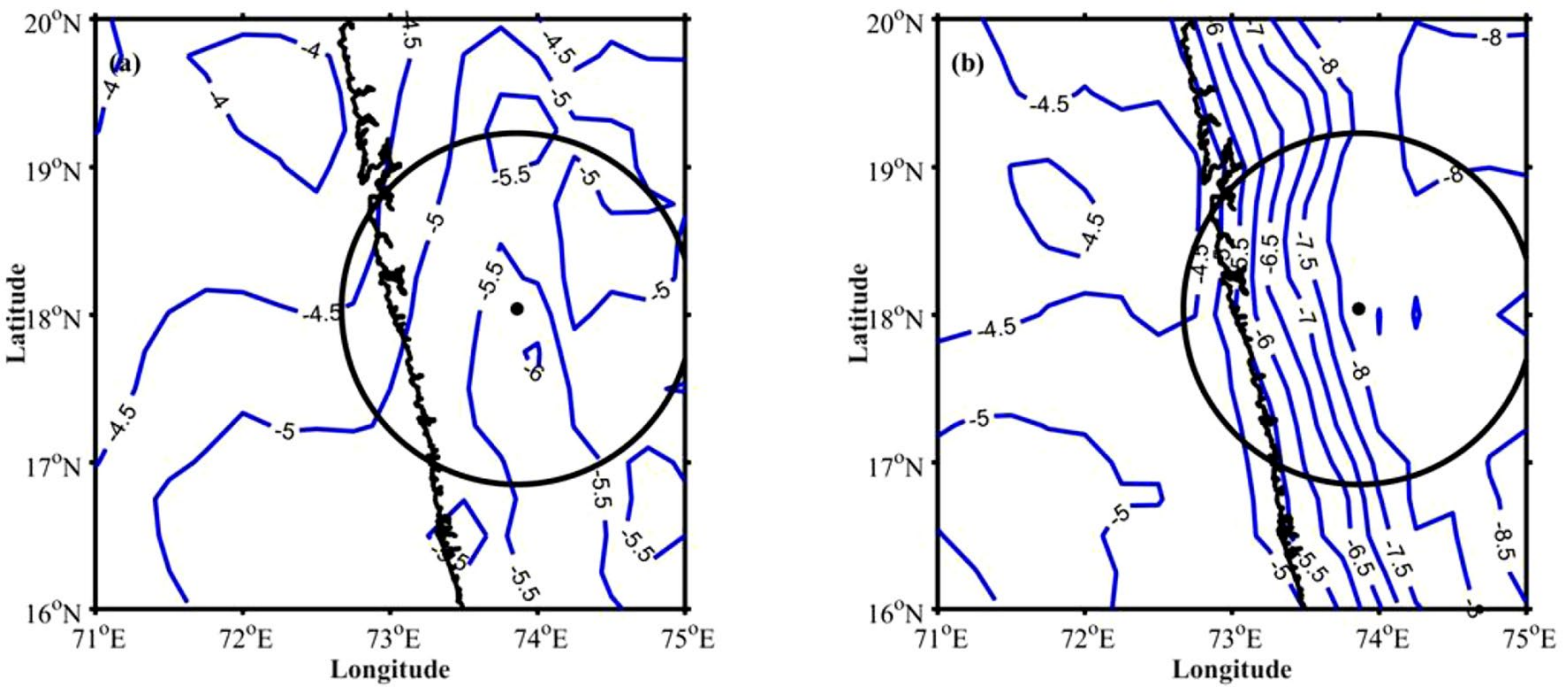
Equivalent potential temperature difference (K) between 500 and 850 hPa levels during (**a**) 0000–0300 LT and (**b**) 1200–1500 LT.

It can be observed from Fig. 2c,d that the CSs developed during afternoon hours are deeper and more intense than those developed in the nighttime. What are the underlying physical processes that are responsible for the development of deeper CSs during afternoon hours? The moistening of the mid-troposphere by large-scale dynamical processes can lead to deeper CSs^50,51^. For this, the moistening of the mid-troposphere before the development of deep convection, known as pre-conditioning, plays a crucial role. The other possible mechanisms including cold pools, downdraft strength, MCS strength and lifecycle etc. can also be equally important in understanding the observed convective activity peak. For example, Feng et al.^52^ showed the importance of isolated and intersecting cold pools in the initiation and sustenance of deep convection. The examination of these factors in observed CSs variability is not in the scope of this work. In this study, we explored the role of mid-tropospheric moistening in the development of deep convection over the high altitude regions of WG. To elucidate this, the amount of water vapour present in the troposphere during 0000–0300 and 1200–1500 LT (pre-conditioning) is analysed over the radar domain. Figure 7a shows the longitude-height (in pressure coordinates) cross-section of specific humidity difference between pre-storm conditions (1200–1500 LT and 0000–0300 LT). Here, the positive values of specific humidity indicate higher moisture availability during afternoon hours (1200–1500 LT), and negative values indicate the moistening during nighttime (0000–0300 LT). The specific humidity is higher in the lower troposphere (up to 700 hPa) during nighttime over oceanic regions. The vertical wind (Fig. 7b) shows convergence in the lower troposphere (up to 700 hPa) during nighttime. In addition, the difference in equivalent potential temperature between 500 and 850 hPa during 0000–0300 LT (Fig. 8a) shows that the gradient is relatively smaller. Further, it is observed that the mid-tropospheric winds are coming from continental regions (Figure not shown), which brings cold and dry air to the radar domain. The entrainment of dry ambient air into the mid-troposphere can play a vital role in limiting the vertical extent of CSs during early morning hours^51,53^. Whereas the vertical velocities are higher during 1200–1500 LT, and higher specific humidity is found up to 400 hPa along the upslope regions and on higher altitudes in the afternoon hours. This indicates the moistening of the mid-troposphere during afternoon hours over higher altitudes and along the mountain’s upslope regions. The equivalent potential temperature shows a highly unstable environment in the mid-troposphere along the upslope regions and on the mountain top during the afternoon (Fig. 8b). Recently, Krishna et al.^54^ derived convective available potential energy (CAPE) using INSAT-3D observations and showed a bimodal distribution over WG and adjoining ocean with maximum CAPE during afternoon hours. The higher moisture availability in the lower and mid-troposphere, upward motion, and unstable atmosphere in the mid-troposphere tend to develop the CSs deeper during afternoon hours. The dry air in the mid-troposphere, less vertical motion, results in shallow CSs over the coastal and oceanic regions during early morning hours.

## Summary

This study examines the diurnal cycle of CSs over WG and associated large-scale atmospheric conditions, using high spatial and temporal resolution data from the ground-based X-band radar, TRMM-PR and ERA5 reanalysis datasets. The analysis shows a prominent diurnal cycle of CSs over WG and adjoining oceans. The key findings of the present study are:

- CSs show a clear diurnal cycle over different topographic regions. A prominent bimodal distribution in the CS occurrence is observed along the coastal regions, and a weak bimodal distribution on the mountain peak, however, their frequency is different. The afternoon peak is stronger over high altitude regions, whereas the early morning peak is stronger over coastal regions.
- The afternoon maximum (over high altitude regions) is related to local topographical lifting, likely aided by solar heating. The early morning peak over coastal regions is induced by the low-level convergence between the prevailing monsoon winds and the thermally induced downslope winds.
- During afternoon hours, the observed CSs are deeper than those developed during early morning hours. While orography is the primary mechanism for the lifting of moist air, the underlying thermodynamics also play an important role in developing deeper and intense CSs during afternoon hours.

Even though the present study utilized only one season of data collected from ground-based X-band radar, the results are worth studying because such high-spatial-temporal resolution measurements can reasonably represent the background dynamical and thermodynamical mechanisms.

## Methods

### Data

The present study utilized data (June–September of 2014) collected from ground-based X-band radar (operating frequency is $\sim$ 9.535 GHz) deployed at Mandherdev (18.04$^\circ$ N, 73.86$^\circ$ E, and $\sim$ 1.3 km above mean sea level), a remote location on the hilltop of WG, India. The X-band radar was manufactured by Enterprise Electronics Corporation (EEC), Alabama, USA. The X-band radar is a scanning radar and mounted on a truck-trailer for mobile use. The radar has a parabolic horn-type antenna of diameter 2.4 m and a half-power beamwidth of 0.97$^\circ$. The radar performs 3D scan of the atmosphere in 360$^\circ$ azimuth with an angular resolution of 0.5$^\circ$, and 18 constant elevation (0.5$^\circ$ to 90$^\circ$) and repeating at every 12 min. The radar signals are processed using Enigma III^+^ signal processor. Detailed technical specifications can be found in Das et al.^38,55^. Routine calibrations are performed for transmitted power and frequency, pulse width, received power, gain, and dynamic range as per the standard procedures.

To compare the diurnal cycle of CS occurrence, the precipitation radar (PR) onboard Tropical Rainfall Measuring Mission (TRMM) satellite measurements are used. Even though the operating frequency of TRMM-PR ($\sim$ 13.8 GHz) is different from ground-based X-band radar ($\sim$ 9.535 GHz), the climatology of CS frequency from the remote sensing platforms would help to improve retrieval algorithms. In this regard, 11-seasons (1999–2006 and 2010–2012) of TRMM-PR reflectivity measurements (2A25 product) are used to find the CS occurrence over the radar domain. The reflectivity threshold of 35 dBZ at 2 km is considered to identify CSs within each pixel of the TRMM-PR measurements.

To explore the plausible mechanism for the observed diurnal cycle of CSs, the latest global reanalysis data, European Centre for Medium-Range Weather Forecasts (ERA5; Copernicus Climate Change Service, C3S), is used. The ERA5 data are available at 0.25$^\circ$ spatial resolution and 1-h temporal resolution. The ERA5 reanalysis provides the atmospheric parameters, such as temperature, humidity, winds, etc., at 37 vertical pressure levels from 1000 to 1 hPa. The present study utilized temperature, specific humidity, horizontal and vertical winds, divergence at the surface and different pressure levels. As the spatial and temporal resolutions of the reanalysis data are coarser compared to ground-based radar, the true representation of background physical mechanisms are questionable. Hence, to understand how the ERA5 can describe the atmospheric parameters in such a hilly and uneven topography, the near-surface temperature over 17.5–18$^\circ$ N, and 73.5–74$^\circ$ E region are compared with surface temperature measurements collected from the automatic weather station (AWS) at Mahabaleshwar (17.92$^\circ$ N, 73.6$^\circ$ E, $\sim$ 1.4 km AMSL) in WG. The diurnal variability in near-surface temperature from ERA5 and AWS is shown in Supplementary Figure (Fig. S1), and the monthly variability is shown in Fig. S2. It can be observed that the reanalysis data reasonably represented the diurnal and monthly variability in near-surface temperature over the radar domain, however, the magnitude is different. So the ERA5 data can adequately be used to understand the physical processes controlling the diurnal cycle of CSs over WG.

### Analysis

The present study uses a state-of-the-art objective cell tracking algorithm known as Thunderstorm Identification Tracking Analysis and Nowcasting (TITAN) proposed by Dixon and Weiner^56^. To identify the CSs, a threshold value of 35 dBZ is used for radar reflectivity. The other criteria used for storm identification is that the storm should have a minimum volume of 30 km^3^ and should persist for two-volume scans (i.e., 24 min). TITAN storm-tracking algorithm is applied to three-dimensional gridded radar data. TITAN’s output provides various storm properties such as storm centroid position, storm area, volume, height, vertically integrated liquid water (VIL), etc. Further, to examine the diurnal variations in CSs occurrence over WG and adjoining oceans, harmonic analysis is performed on the occurrence frequency of CSs following Wilks^57^. In the present study, the first two harmonics are considered to understand the diurnal and semi-diurnal variations in the CS occurrence.

The present study used only one season (June–September 2014) data to understand the diurnal cycle of CSs over WG. The unavailability of the long-term dataset is one of the caveats of this work as the large-scale atmospheric conditions may be different for a particular year. For example, the Indian summer monsoon (ISM) rainfall was deficient during 2014 due to the El Nino and negative Indian Ocean dipole conditions that prevailed prior to and early part of the monsoon^58^. The ENSO-ISM teleconnections may have an impact on the physical mechanisms that govern the diurnal cycle. Rauniyar and Walsh^59^ studied the diurnal cycle of rainfall over Australia during El Nino and La Nina phases. They found an increase (decrease) in rainfall during La Nina (El Nino) periods. However, the diurnal cycle of rainfall remains the same. Hence, the ENSO-ISM teleconnections can modulate the strength of the diurnal cycle of CSs, but not the variability. In addition, the use of reanalysis data having different spatial resolution compared to the radar observation may have deficiencies that do not allow the true representation of the large scale environment responsible for the diurnal cycle of CSs, especially in the complex terrain of WG. Though the spatial averaging of large-scale environment from the ERA5 data may not represent the storm-scale convection, however, the ERA5 is the most comprehensive and high-resolution reanalysis available currently and can be used to understand the physical mechanism of the observed diurnal cycle of CSs over WG. With these caveats, the authors interested to understand the diurnal cycle of CSs over WG.

## Supplementary Information

Supplementary Figure S1.

Supplementary Figure S2.
