# Supplementary figures and images for: Physical processes controlling the diurnal cycle of convective storms in the Western Ghats
Source: Sci Rep. 2021 Jul 8;11:14103. doi: 10.1038/s41598-021-93173-0 (PMC8266914; doi:10.1038/s41598-021-93173-0)

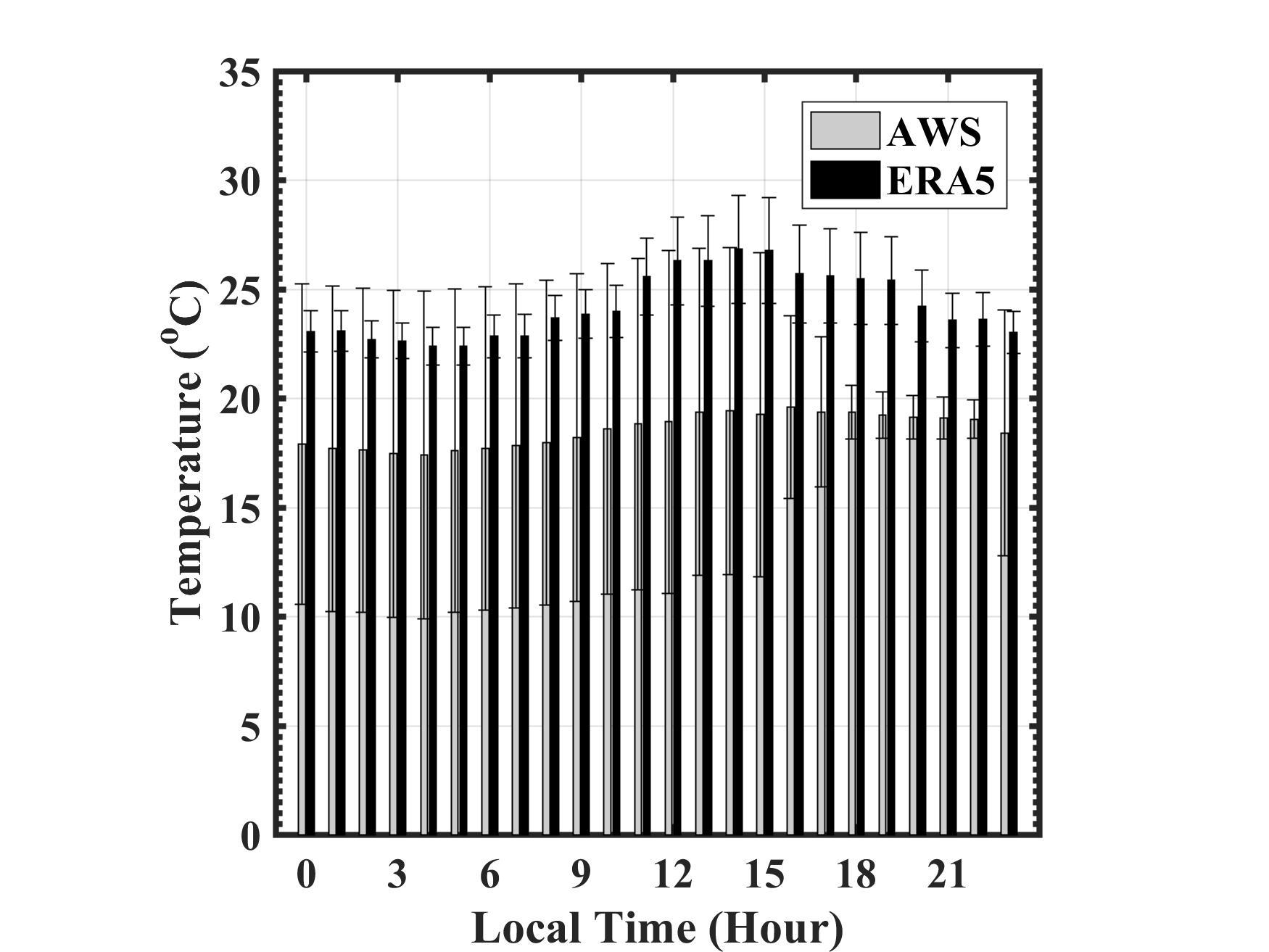

Supplement: Supplementary file 1 — Supplementary Figure S1. [file 41598_2021_93173_MOESM1_ESM.jpg]

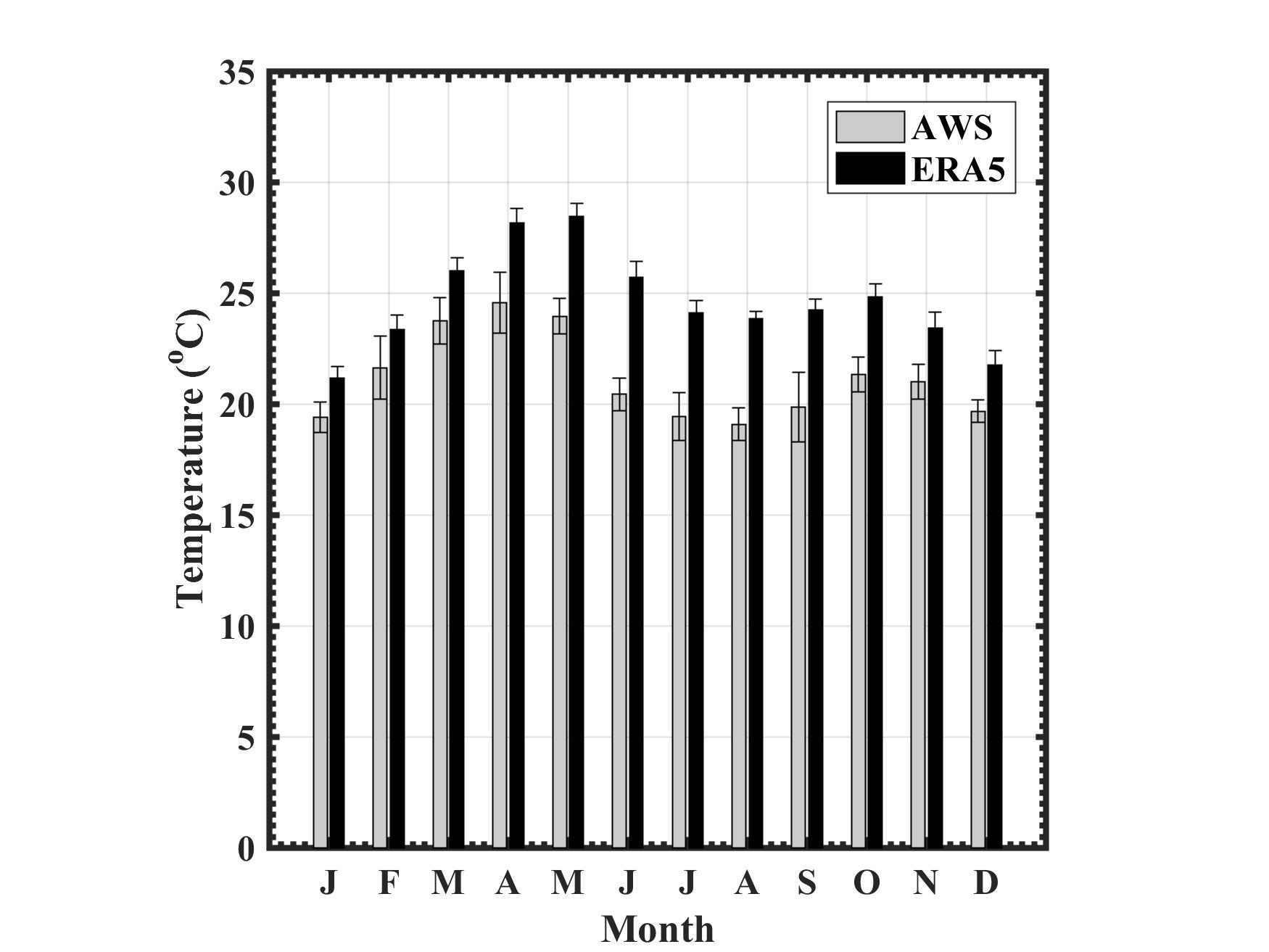

Supplement: Supplementary file 2 — Supplementary Figure S2. [file 41598_2021_93173_MOESM2_ESM.jpg]
